# Supplementary material for: Single-cell chromatin accessibility and lipid profiling reveals SCD1-dependent metabolic shift in adipocytes induced by bariatric surgery
Source: PLoS One. 2021 Dec 31;16(12):e0261783. doi: 10.1371/journal.pone.0261783 (PMC8719700; doi:10.1371/journal.pone.0261783)
Supplement: S1 Fig — (DOCX) [file pone.0261783.s001.docx]

**
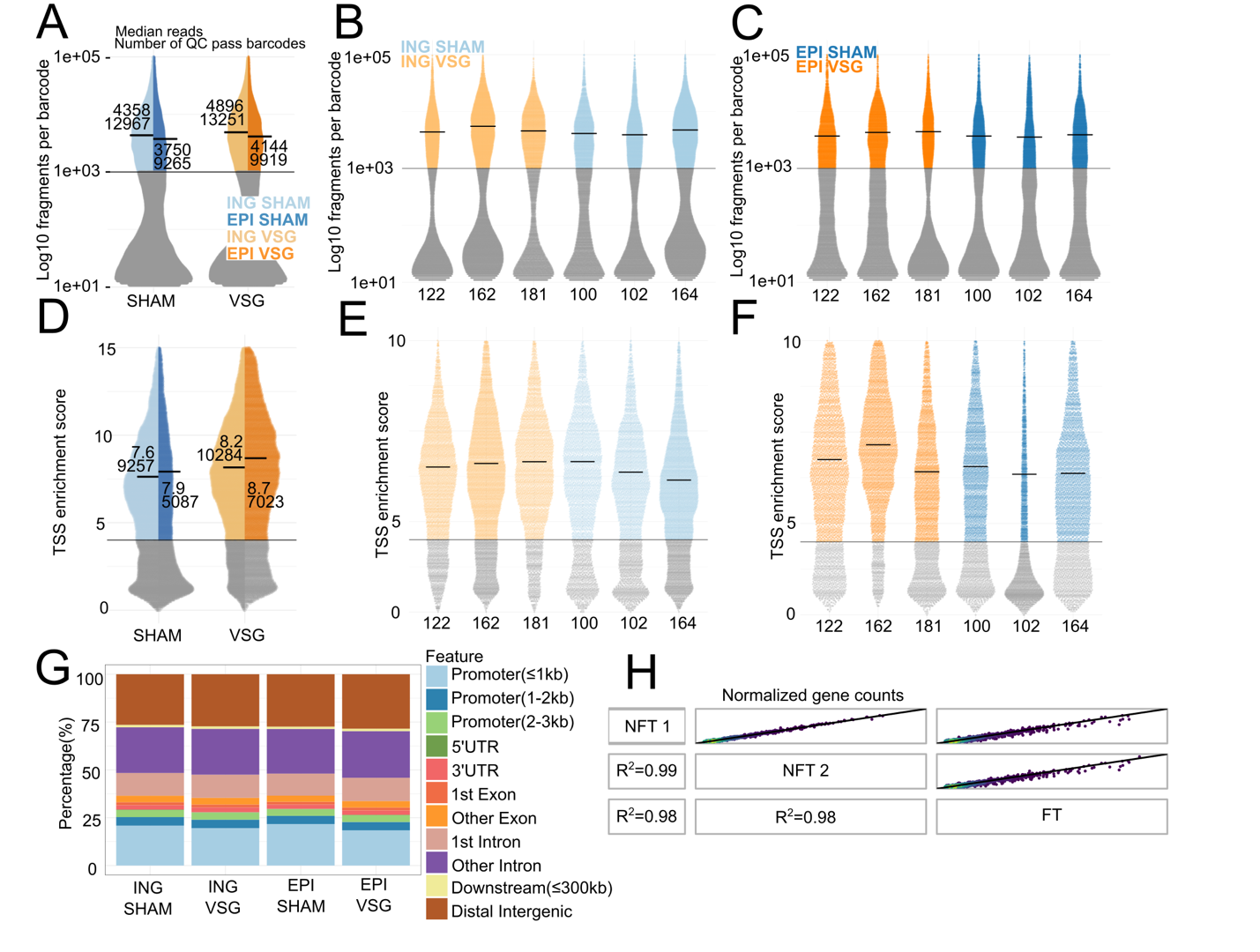
Supplementary Figure 1. Quality control metrics for each biological replicate**

A. Fragment count distributions per barcode for each cell from SHAM- or VSG-derived depots. Cells with 1,000 or more fragments were kept for further analysis. The median fragment count for each group (top number) is indicated with a thick bar; lower number identifies the number of bar codes or cells passing that cutoff. B, C. Fragment count distributions per barcode for each cell for each biological replicate from ING (B) and EPI (C). The median value for each group is shown with a thick bar. D. TSS enrichment score analysis of cells with 1,000 or more fragments per cell. Cells with a score of 4 or higher were kept for further analysis. The median score for each group (top number) is indicated with a thick bar; lower number identifies the number of bar codes or cells passing that cutoff. E, F. TSS enrichment score per barcode for each cell for each biological replicate ING (E) and EPI (F). Numbers below violin plots denote the individual mouse identification number. H. Spearman correlation of normalized gene counts comparing non-freeze thaw technical replicate libraries (NF1 and NF2) vs. the freeze-thaw library (FT) from the same pool of tagmented nuclei.
