# Supplementary material for: Single-cell chromatin accessibility and lipid profiling reveals SCD1-dependent metabolic shift in adipocytes induced by bariatric surgery
Source: PLoS One. 2021 Dec 31;16(12):e0261783. doi: 10.1371/journal.pone.0261783 (PMC8719700; doi:10.1371/journal.pone.0261783)
Supplement: S2 Fig — (DOCX) [file pone.0261783.s002.docx]

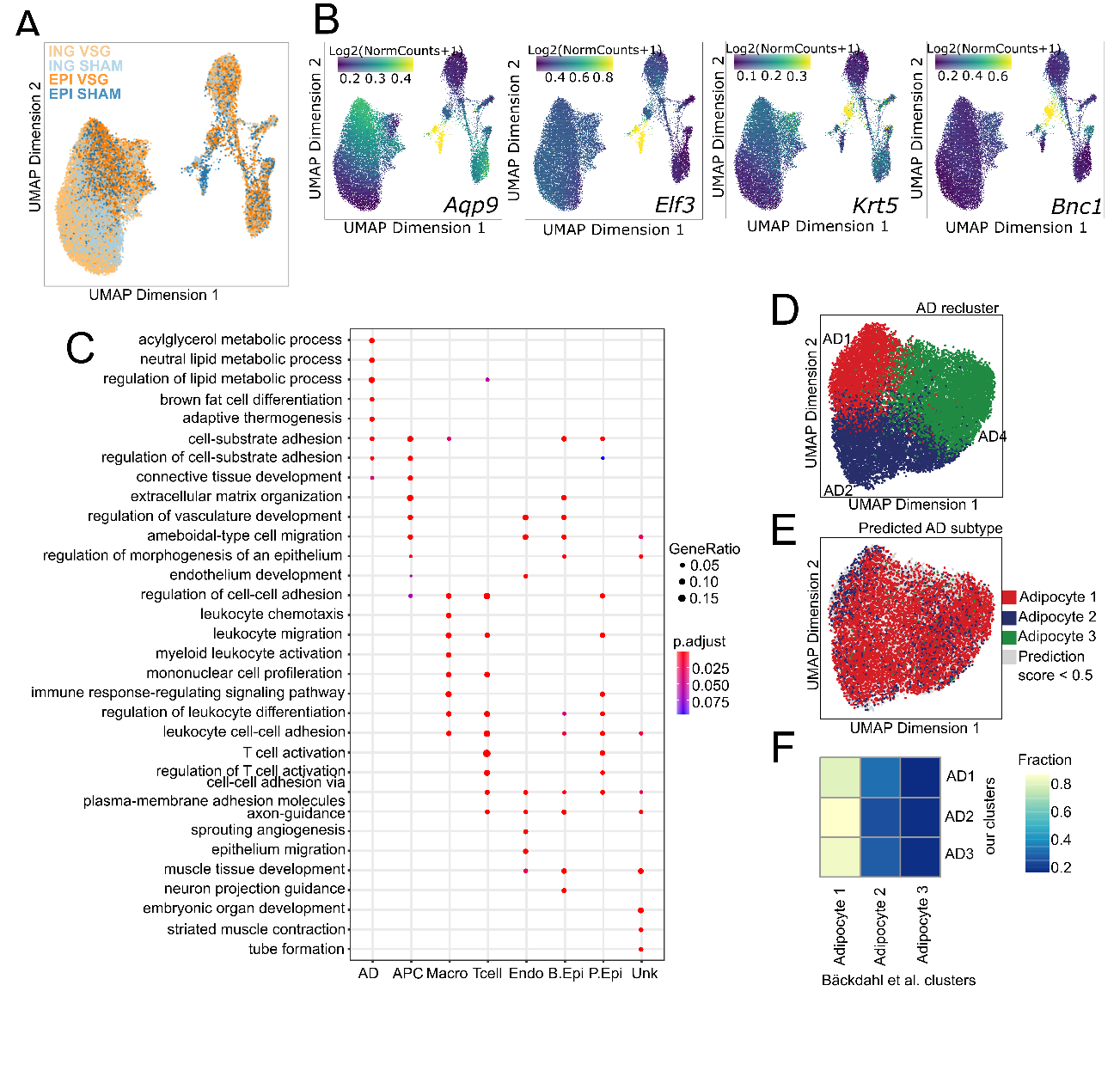


**Supplementary Figure 2. Cell type assignment of clusters**

A. UMAP plot showing distribution of cells from inguinal (light blue, ING-SHAM; light orange, ING-VSG) and epididymal (dark blue, EPI-SHAM; dark orange, EPI-VSG).

B. UMAP with imputed accessibility of basal epididymal (*Aqp9* and *Bnc1)* and principal epididymal (*Krt5* and *Elf3)* cell type markers.

C. GO enrichment for top 500 DA genes from each major cluster.

D. UMAP plot of reclustered adipocyte subgroups. AD1 (red), AD2(blue), and AD4 (green).

E. UMAP plot of predicted adipocyte subtypes through integration with spatial transcriptomics dataset of human adipose tissue (spatial transcriptomics data from Bäckdahl et al.)

F. Fraction of our adipocyte subclusters (AD1,2,4) predicted by integration with spatial transcriptomics dataset
