# Supplementary material for: Single-cell chromatin accessibility and lipid profiling reveals SCD1-dependent metabolic shift in adipocytes induced by bariatric surgery
Source: PLoS One. 2021 Dec 31;16(12):e0261783. doi: 10.1371/journal.pone.0261783 (PMC8719700; doi:10.1371/journal.pone.0261783)
Supplement: S3 Fig — (DOCX) [file pone.0261783.s003.docx]

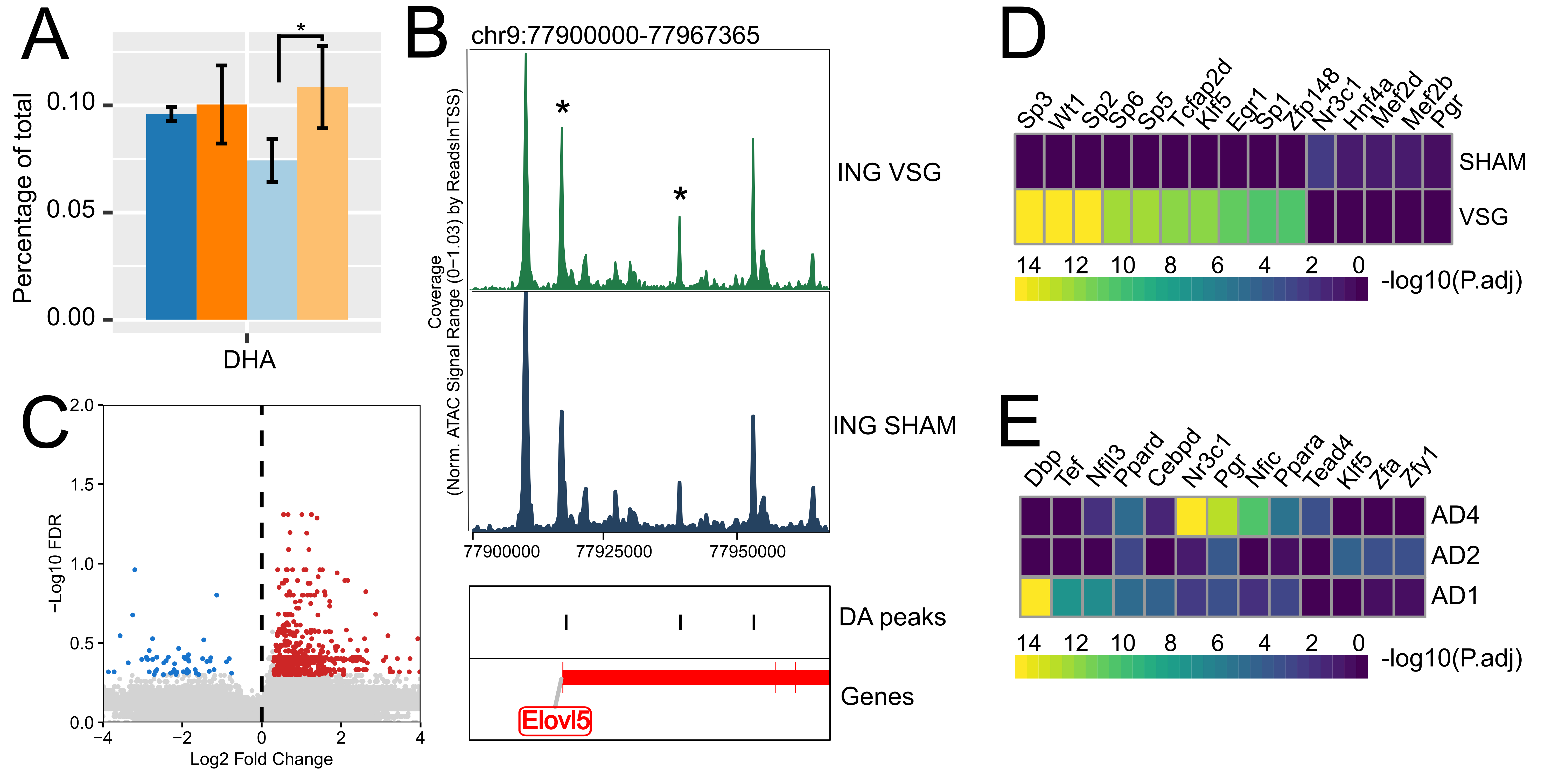


**Supplementary Figure 3. Differential accessibility of transcription factor motifs and changes in DHA fatty acid in response to VSG**A. Percent of DHA fatty acid in adipose tissues. (*n*=3)
B. Genome browser shot of the promoter region of *Elovl5* showing STAT5A motifs (denoted by *) within peaks that are more accessible in VSG. Differentially accessible, DA.
C. Differential accessible peaks in EPI adipocytes. There were 593 peaks more accessible in EPI VSG (positive fold change) and 66 peaks more accessible in EPI SHAM (negative fold change).
D. Enriched transcription factor motifs in differentially accessible peaks in EPI adipocytes.

E. Enriched transcription factor motifs in differentially accessible peaks comparing AD1,AD2, and AD4 in SHAM ING.
Differentially accessible, DA; DHA, Docosahexaenoic acid
