# Supplementary material for: Single-cell chromatin accessibility and lipid profiling reveals SCD1-dependent metabolic shift in adipocytes induced by bariatric surgery
Source: PLoS One. 2021 Dec 31;16(12):e0261783. doi: 10.1371/journal.pone.0261783 (PMC8719700; doi:10.1371/journal.pone.0261783)
Supplement: S4 Fig — (DOCX) [file pone.0261783.s004.docx]

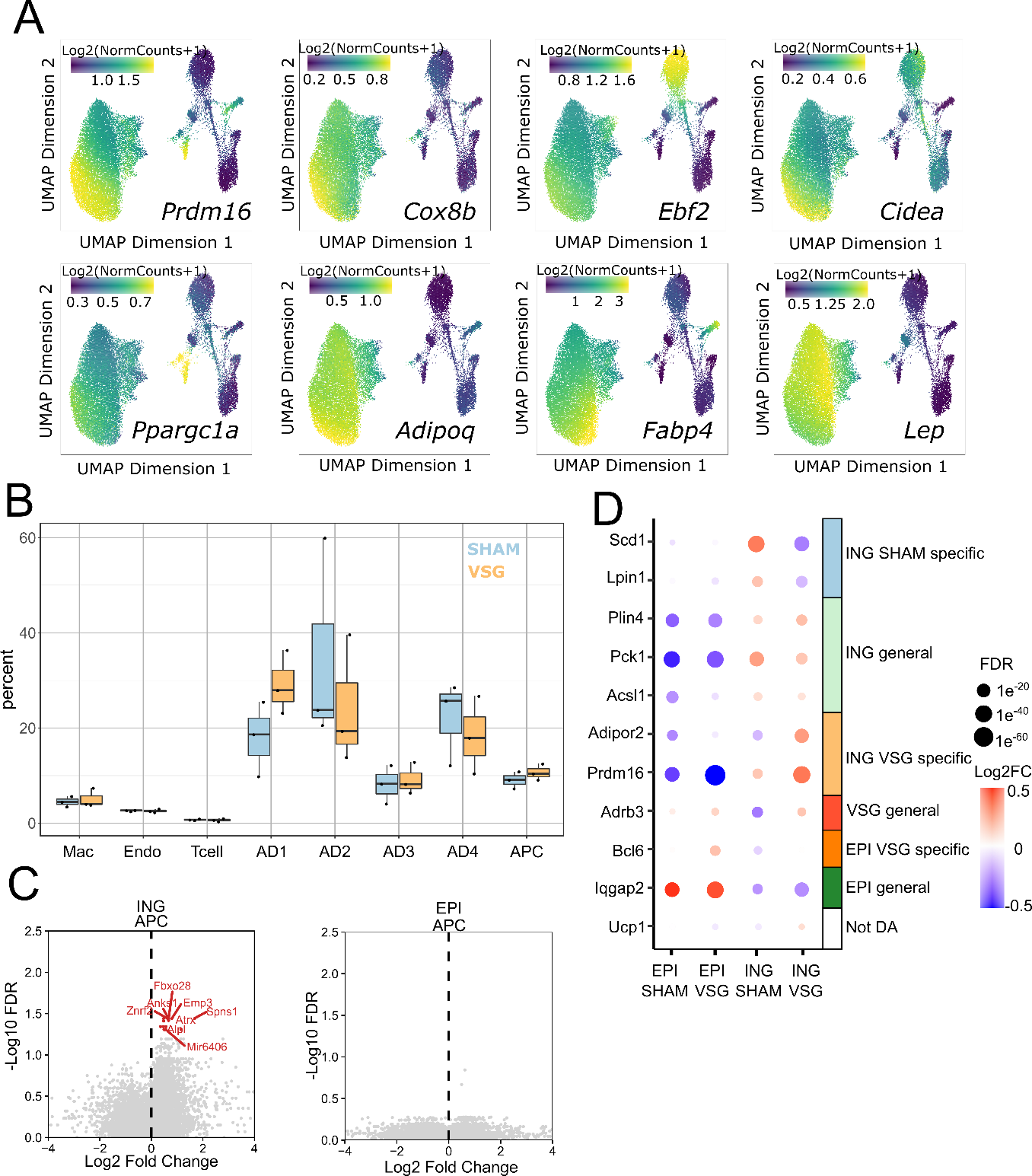
**Supplementary Figure 4. Differential accessibility of genes response to VSG in the ING depot**A. Imputed marker gene accessibility using MAGIC overlaid on the UMAP plot for beige-associated adipocyte markers (*Prdm16, Cox8b, Ebf2, Cidea, Ppargc1a*) and white-associated adipocyte markers (*Adipoq, Fabp4, Lep*).

B. Relative distribution of cell types in the ING depot from SHAM and VSG animals. Percentages were normalized to the total number of cells within each biological replicate. (*n*=3)

C. Gene accessibility differences between VSG (positive fold change) and SHAM (negative fold change) in adipocyte progenitor cells (APC) of ING (left) and EPI (right).

D. Relative average accessibility of genes with depot specific responses to VSG.
