# Supplementary material for: Single-cell chromatin accessibility and lipid profiling reveals SCD1-dependent metabolic shift in adipocytes induced by bariatric surgery
Source: PLoS One. 2021 Dec 31;16(12):e0261783. doi: 10.1371/journal.pone.0261783 (PMC8719700; doi:10.1371/journal.pone.0261783)
Supplement: S1 File — (DOCX) [file pone.0261783.s005.docx]

**Supporting Information**

**Freezing nuclei after tagmentation**

Batch effects are a significant concern in single-cell sequencing experiments and can arise when nuclei extractions, tagmentation, library amplifications are spread across multiple days. We assessed a protocol alteration to minimize batch effects by consolidating library preparation steps in fewer days. We cryopreserved nuclei after tagmentation to allow samples to be prepared in tandem. Though cryopreservation of cells for bulk ATAC-seq did not lead to reduced quality data, this has not yet been tested for single-cell ATAC-seq [1]. Notably, compared to freshly isolated nuclei, frozen nuclei showed no discernable differences in quality with a high degree of correlation in counts per gene (Fig S1E). We applied this strategy to our adipose snATAC-seq library preparations, increasing the per day throughput of each library preparation step.

**References**

1. Milani P, Escalante-Chong R, Shelley BC, et al. Cell freezing protocol suitable for ATAC-Seq on motor neurons derived from human induced pluripotent stem cells. *Sci Rep*. 2016;6(1):1-10. doi:10.1038/srep25474
